# Analysis of 13000 unique *Citrus *clusters associated with fruit quality, production and salinity tolerance

**DOI:** 10.1186/1471-2164-8-31

**Published:** 2007-01-25

**Authors:** Javier Terol, Ana Conesa, Jose M Colmenero, Manuel Cercos, Francisco Tadeo, Javier Agustí, Enriqueta Alós, Fernando Andres, Guillermo Soler, Javier Brumos, Domingo J Iglesias, Stefan Götz, Francisco Legaz, Xavier Argout, Brigitte Courtois, Patrick Ollitrault, Carole Dossat, Patrick Wincker, Raphael Morillon, Manuel Talon

**Affiliations:** 1 Centro de Genomica, Instituto Valenciano de Investigaciones Agrarias. Carretera Moncada – Náquera, Km. 4.5 Moncada (Valencia) E46113, Spain; 2 BET-ITACA, Universidad Politécnica de Valencia, Camino de Vera, s/n 46022 Valencia, Spain; 3 CIRAD AMIS, UMR PIA – Avenue Agropolis – TA 40/03 34398 Montpellier Cedex 5, France; 4 Genoscope-Centre National de Séquençage and Centre National de la Recherche Scientifique (CNRS) Unité Mixte de Recherche (UMR)-8030, 91000 Evry, France; 5 CIRAD FLHOR, UPR "Amélioration génétique d'espèces à multiplication végétative", Avenue Agropolis – TA 40/03 34398 Montpellier Cedex 5, France

## Abstract

**Background:**

Improvement of *Citrus*, the most economically important fruit crop in the world, is extremely slow and inherently costly because of the long-term nature of tree breeding and an unusual combination of reproductive characteristics. Aside from disease resistance, major commercial traits in *Citrus *are improved fruit quality, higher yield and tolerance to environmental stresses, especially salinity.

**Results:**

A normalized full length and 9 standard cDNA libraries were generated, representing particular treatments and tissues from selected varieties (*Citrus clementina *and *C. sinensis*) and rootstocks (*C. reshni, and C. sinenis *× *Poncirus trifoliata*) differing in fruit quality, resistance to abscission, and tolerance to salinity. The goal of this work was to provide a large expressed sequence tag (EST) collection enriched with transcripts related to these well appreciated agronomical traits. Towards this end, more than 54000 ESTs derived from these libraries were analyzed and annotated. Assembly of 52626 useful sequences generated 15664 putative transcription units distributed in 7120 contigs, and 8544 singletons. BLAST annotation produced significant hits for more than 80% of the hypothetical transcription units and suggested that 647 of these might be *Citrus *specific unigenes. The unigene set, composed of ~13000 putative different transcripts, including more than 5000 novel *Citrus *genes, was assigned with putative functions based on similarity, GO annotations and protein domains

**Conclusion:**

Comparative genomics with Arabidopsis revealed the presence of putative conserved orthologs and single copy genes in *Citrus *and also the occurrence of both gene duplication events and increased number of genes for specific pathways. In addition, phylogenetic analysis performed on the ammonium transporter family and glycosyl transferase family 20 suggested the existence of *Citrus *paralogs. Analysis of the *Citrus *gene space showed that the most important metabolic pathways known to affect fruit quality were represented in the unigene set. Overall, the similarity analyses indicated that the sequences of the genes belonging to these varieties and rootstocks were essentially identical, suggesting that the differential behaviour of these species cannot be attributed to major sequence divergences. This *Citrus *EST assembly contributes both crucial information to discover genes of agronomical interest and tools for genetic and genomic analyses, such as the development of new markers and microarrays.

## Background

Citrus fruits are the first fruit crop in international trade in terms of economic value (FAO, 2004). Citrus fruits are typically grown in 140 countries located in tropical and subtropical areas with "Mediterranean" type climates, often facing severe abiotic stresses such as salinity, drought and iron chlorosis. *Citrus *species also suffer from different diseases and pests that considerably affect tree growth and fruit crop. The survival of the citrus industry is today critically dependent on genetically superior cultivars. However, citrus improvement through traditional techniques is unfortunately very difficult due to the unusual combination of biological characteristics of *Citrus *species, their low genetic diversity and the long-term nature of tree breeding. Thus, *Citrus *show many biological characteristics such as gametophytic self- and cross-incompatibility, apomixy, juvenility, heterozygosis, dormancy, and surprising root/shoot interactions, that strongly hamper Citrus breeding. On the other hand, genetic and allelic diversity in *Citrus *cultivars is very scarce. The global linkage disequilibrium in the cultivated citrus that probably originated from three major taxa, may be the result of an initial allopatric evolution and further limitation for predominant apomixy [1]. The fact that only mutational and/or epigenetic events are apparently involved in the diversification of secondary species, combined with human selection, have strongly reduced global genetic diversity, restricting opportunity for genetic advance.

Genomics has provided new tools for crop improvement, helping to identify and select candidate genes responsible of agronomic characters of interest, and allowing the development of fast methods to incorporate these characters into crop plants. After the completion of the Arabidopsis genome sequence [2] and the publication of sequences of indica [3] and japonica [4] rice, plant researchers have been able to scan these genomes to identify and compare genes of interest. The completion of the poplar genome sequence [5] will supply a model for tree life forms.

EST sequencing projects have facilitated appropriate strategies for gene discovery [6], molecular markers identification [7,8], and many other functional genomic developments and tools, e.g. microarray approaches [9-11]. In *Citrus*, previous EST sequencing projects have released more than 130000 ESTs to Gen Bank, mainly from *C. sinensis*, *C. unshiu *and *C. clementina *[12-16]. This information has been used to develop two different microarray platforms, based on cDNA and short oligo sequences [12,17]. In this work, main efforts have been specifically focussed on the study of pivotal traits for *Citrus *breeding, such as fruit quality, productivity and salinity tolerance. In citrus there are many aspects of fruit quality such as fruit size, shape, colour, texture, flavour and aroma compounds, and several other organoleptic properties that are acquired during ripening and earlier stages of growth [18-20]. Regarding productivity, pivotal traits to be improved are the capacity for fruit set and the resistance to abscission. Clementine mandarin (*Citrus clementina*), for example, is a self-incompatible cultivar that shows elevated ovary and fruitlet abscission while sweet orange varieties (*Citrus sinensis*), that in general exhibit standard fruit-set ratios [21,22], may lose most of the yield during ripening. In addition, the quantity and quality of water can become a limiting factor to economically viable production. In *Citrus*, it is notorious for example, that during the periods of drought, leaves and fruits remain attached to the tree until water is available and soon afterwards these organs abscise [23,24]. It is also well known that salt excess affects the size and quality of the production. The capability of *Citrus *to tolerate salinity is mostly related to the ability of the rootstock to exclude chloride, although the nature of this mechanism remains unresolved [25]. Tolerant Cleopatra mandarin rootstock (*C. reshni*), for example, accumulated lower chloride amounts than sensitive Carrizo (*C. sinenis *× *Poncirus trifoliata*) [26,27]. The scion variety also plays a role in salinity damage, and more tolerant varieties such as Clementine are generally preferred to sweet oranges [28]. Toward this goal, cDNA libraries were prepared from the following selected genotypes: the varieties *Citrus clementina *(cv. Clementina de Nules; elevated fruit quality, low setting) and *C. sinensis *(cv Washington Navel and Navelina; pre-harvest abscission and low salt tolerance, respectively), and the rootstocks *C. reshni *(cv *Cleopatra*; salt tolerant), and *C. sinenis *× *Poncirus trifoliata *(cv Carrizo; salt sensitive). The information generated with this effort, complementary to the Spanish *Citrus *Functional Genomics Project [12], has also been used for the construction of a second generation cDNA microarray of recent release. In the study, special attention has been paid on the methodological aspects, in order to obtain accurate estimates of the number of different transcripts and precise predictions of their function.

The results of this joint initiative of the IVIA, CIRAD and Genoscope designed to provide new information and useful tools for *Citrus *improvement, will speed up the discovery of genes of major agronomic interest and facilitate the development of new markers and methods to rapidly identify improved genotypes.

## Results

### Library Construction

Samples were harvested from 4 different *Citrus *species: the varieties *Citrus clementina *(cv. Clementina de Nules; elevated fruit quality, low setting) and *C. sinensis *(cv Washington Navel and Navelina; pre-harvest abscission and low salt tolerance, respectively), and the rootstocks *C. reshni *(cv *Cleopatra*; salt tolerant), and *C. sinenis *× *Poncirus trifoliata *(cv Carrizo; salt sensitive).

EST sequences were derived from 9 cDNA libraries constructed with standard methods and 1 additional normalized full length cDNA library (Table 1). Standard libraries, prepared from all five cultivars, were designed to represent three main agronomic traits of interest for *Citrus *improvement, i.e. fruit quality, production and salt tolerance. Since fruit quality characteristics are acquired not only during ripening but also along fruit growth [18], 2 libraries covering all pivotal developmental stages were constructed from fruit pulp and peel tissues of the high quality variety, Clementine (FruitTF, and PhII-IIIVesicles1). Abscission zones and surrounding tissues from developing ovaries, fruitlets, leaves and ripe fruits were represented in 4 libraries (AbsAOv1, Abs Cov1, AbsDev, and AbsCFruit1) from Clementine, the genotype with impaired fruit set [29] and Washington Navel, the variety with higher pre-harvest abscission. To study the response to abiotic stresses, 3 libraries were prepared with samples derived from leaves and roots subjected to salinity or dehydration (LSH, KCl-Salt1, and EHR) from the salt tolerant rootstock, Cleopatra, the sensitive hybrid, Carrizo and the sensitive scion, Navelina [27,28].

The full length library was constructed in Clementine plants, the species of main interest, grown in open field and in controlled greenhouse conditions. A broad variety of tissues and organs at different developmental stages were harvested from healthy plants and from plants subjected to many biotic (viruses, insects, fungus...setc) and abiotic (drought, salinity, ozone, alkaline-calcareous soils, flooding.etc) stresses. This strategy was planned to obtain the widest representation of the *Citrus clementina *transcriptome, including low abundant cDNAs, and to facilitate identification of genes of agronomic interest. A detailed description of the libraries is given in Materials and Methods.

### EST Sequencing and Clustering

**Figure 1:**
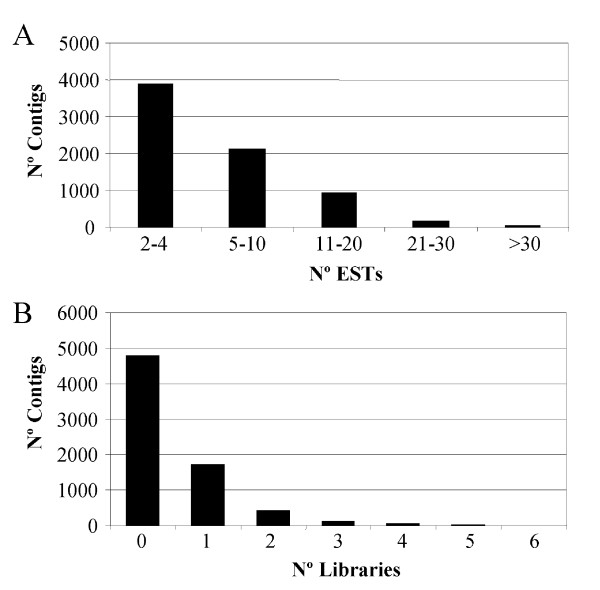
**EST assembly results**. A – Distribution of ESTs incontigs. B – Number of contigs containing ESTs from 1 or more cDNA libraries.

A total number of 54136 clones were single-pass sequenced from their 5' end. After low quality and vector trimming, 52626 sequences longer than 100 bp were assembled with the CAP3 program [30]. All these sequences are available at the EST section of the GenBank. The assembly grouped 44082 ESTs into 7120 contigs, while 8544 sequences remained as singletons. The combined set of contigs and singletons resulted in 15664 unigenes representing different putative transcripts from *Citrus *species. The average length of the unigenes was 1071 bp, and 9877 of them (63%) were longer than 1000 bp. The distribution of ESTs in the contigs was the expected, with many clusters composed of 10 or less ESTs (Fig 1A). To estimate redundancy levels, the 15664 unigenes were compared with each other with the BLASTN program [31]. Sequences with at least 98% nucleotide identity over a minimum of 100 bp were assumed to be derived from the same transcript and therefore were clustered in *supercontigs *using an in-house R algorithm. Clustering of the unigenes resulted in 1135 *supercontigs *and 12759 singletons, indicating ~26% redundancy. Thus, the real number of identified expressed genes was close to 13900.

The unigene consensus sequences were used as queries in a BLASTN search against a database including 130400 ESTs from *Citrus *species retrieved from GenBank. An e value of 1e-13 was used as a cut off to ensure that only almost identical sequences were detected. The results showed that more than 5159 unigenes (33%) did not produce a significant hit, indicating that these sequences had not previously been described in *Citrus *EST collections. However, the possibility that other parts of the same parental genes were present in these collections can not be discarded. Most novel sequences (4673) were derived from the normalized library and might represent transcripts expressed at very low levels. This idea was supported by the fact that 75% of these sequences were singletons. On the other hand, no major divergences or differences at the sequence level were observed between all *Citrus *species analyzed, including the five cultivars used in this study and those with sequences submitted to GenBank (*C. sinensis, Citrus *× *paradisi*, *and C. unshiu*, mainly).

The analysis of the contigs (Fig 1B) indicated that many of them were composed of ESTs from a single library (67%), while only 195 contigs (2%) included ESTs from 4 or more libraries. The unigene Contig6498 that displayed high similarity with ATPase-like proteins, for example, was found in 7 libraries, although it was composed of only 9 ESTs. The number of exclusive unigenes of a given cDNA library was obtained adding the number of singletons and the contigs composed of ESTs from this library (Table 1). It was determined that 11432 (72.9%) clusters were exclusive of a particular library. The standard cDNA libraries provided ~21% of the assembled ESTs while the number of exclusive clusters from these libraries represented more than 26% of the unigenes.

### Sequence Annotation

Annotation of the assembled sequences was initially based on primary sequence homology searches. A BLASTX search performed against the GenBank non redundant protein database [32] with an e value cut off ≤ 1e-10, yielded 13339 unigenes with significant hits. A number of 4541 protein homologs were annotated as unknown, unnamed, hypothetical or expressed proteins.

BLASTX searches were also performed against the complete protein sets of *Arabidopsis thaliana *[33] and *Oryza sativa *[34]. The results were similar, since 12336 and 11996 significant hits were found in Arabidopsis and rice, respectively. BLAST results were parsed, determining for each *Citrus *unigene, the best hit name and description, the extent of the aligned region, the percentage of similarity and the e value. Sequences were classified based on the ORF conservation: 5595 unigenes had very high similarities (80%–100%), 5729 clusters showed high similarities (60 – 80%), while only 1883 unigenes had moderate similarities (40%–60%) and 132 sequences displayed low similarities (30%–40%). No similarities below 30% were obtained. These results indicated that a large number of unigenes (40%) had a very strong match (sequence conservation ≥ 80%) with the top-score hit of the BLASTX results.

The extent of the region of similarity between a given unigene and its best hit protein was also determined, including all High-scoring Segment Pairs (HSPs) for one hit. To this end, the following assumptions were taken: when similarity regions expanded along the complete length of the hit protein, unigenes were supposed to include a complete ORF; if HSPs matched the amino-terminal region of the hit sequence, the cDNA clones from which these ESTs were derived probably contained a complete mRNA partially sequenced; finally, when HSPs located at the carboxy-terminal region of the hit protein, cDNA clones were assumed to correspond to truncated mRNAs. The *Citrus *unigenes were classified according to these criteria, resulting in 4065 complete ORFs, 6082 complete cDNA clones, and 4132 partial or truncated cDNA clones. Taken together the complete ORFs and clones, the *Citrus *EST collection contained at least, 10147 complete cDNA clones.

The species of the best hit sequences produced in the BLASTX search against the non redundant database were registered and classified (data not shown). Not surprisingly, 7725 hits (55.2%) corresponded to *A. thaliana *and 2610 (18.6%) to other eudicots species. About 400 unigenes produced significant best hits from species other than plants (mainly bacteria, fungi, and insects), with a high degree of conservation (= 80%). 298 of these clusters had no significant hits from Arabidopsis or rice, and a BLASTN search performed against the non redundant and EST GenBank databases, confirmed they might be contaminant sequences not originated from *Citrus *species.

**Figure 2:**
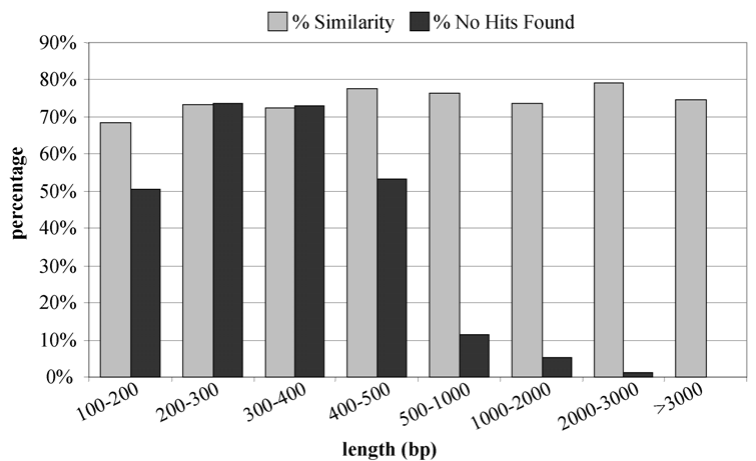
**BLASTX Annotation**. Relationship between the length (bp) and both the number of unigenes producing no hits (black columns) and the average similarity (grey column) with respect the best hits.

A considerable number of sequences (10 to 15%) did not produce a significant hit in the BLAST searches performed. The correlation between the number of sequences that produced no hits and the length of the unigene (Fig 2), showed that 607 out of 954 sequences shorter than 500 bp (63%) did not produce a significant hit, while the percentage was only 7% for longer sequences (1067out of 14710 unigenes). Since the BLASTX searches were performed against protein databases, the high number of no hits found for the clusters shorter than 500 bp, may indicate that these regions did not contain coding sequences, corresponding to mRNA UTRs. The absence of significant hits was certainly not due to low quality of the shorter sequences, because the level of similarity of the unigenes was always high regardless the size of the sequence (Fig 2), indicating that shorter sequences had the same quality than longer ones.

The sequences that did not produce significant hits were used as queries in a BLASTN search performed against the GenBank non redundant nucleotide database [32]. Only 40 unigenes produced significant hits showing high similarity levels (80 – 100%). A total of 24 sequences presented similarity with the complete sequence of *Poncirus trifoliata Citrus *tristeza virus resistance gene locus, the only genomic BAC clone (282699 bp long) from a *Citrus *species that has been sequenced [35].

The same set of unigenes produced 647 significant hits (conserved region longer than 100 bp and similarity ≥ 80%) in a further BLASTN search against the GenBank EST database [32], indicating that similar transcripts were previously isolated. Further analysis showed that most of the hits derived only from *Citrus *species, suggesting that these clusters might be putative *Citrus *exclusive genes.

### Protein translation and annotation

A search for domains associated with a Hidden Markov Model profile was intended to improve annotation of the EST collection. To obtain better templates for annotation, the translation of the unigenes consensus sequences into polypeptide ones was carried out with the prot4EST prediction pipeline, which produces robust translations from EST sequences [36]. A BLASTP search was performed with the polypeptide sequences against the GenBank protein database [32], and 77% of them produced the same hit than the original DNA sequences, showing the accuracy of the translations. All polypeptides shorter than 30 aa, and those shorter than 100 aa without a significant hit were discarded, and the final number of useful protein translations was 14782. The parsing of the BLASTP results confirmed that the unigenes initially classified as complete ORFs coded for complete proteins.

Once protein sequences were obtained and tested, searches against pattern or signature databases were performed. The InterPro database was chosen for these searches, as it unites secondary databases that contain overlapping information on protein families, domains and functional sites [37]. The standalone version of the interProScan tool, that combines the protein function recognition methods of the member databases of InterPro into one application, was used for the analysis. The total number of unigenes that produced hits and the number of different motifs identified varied highly depending on the database size and the analysis method. Motif search against the Pfam database produced the largest number of protein hits (7141) and also identified a high number of different motifs (1666). Table 2 shows the 20 most abundant conserved domains found with Pfam. These motifs were responsible of the 34% of the total hits. Further analysis was carried out on the subset of 2482 proteins predicted as complete with the BLASTX analyses that produced Pfam hits (Table 3). Most of the proteins, 76.15%, displayed a single type of functional domain; proteins combining 2 different motifs accounted for the 20.43%, while polypeptides with 3 or 4 different conserved domains were in a low number (3.42%).

### Gene Ontology Annotation

**Figure 3:**
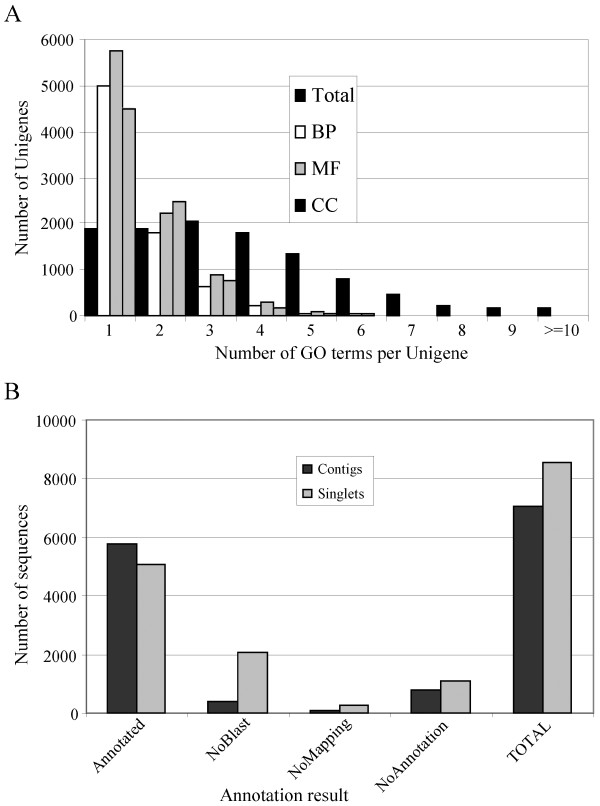
**Gene Ontology Annotation**. A – Intensity of GO annotation. The number of unigenes with GO annotations for each of the main Gene Ontology categories, biological process (BP), molecular function (MF) and cellular component (CC) is shown. The total column includes unigenes with GO terms from the three categories. B – GO annotation in contigs (black columns) and singletons (grey columns). Annotated = sequences with functional GO annotation; NoBLAST = sequences with no BLAST results; NoMapping = sequences that produced BLAST hits without ontology annotations; NoAnnotation = sequences that produced BLAST hits with not significant ontology annotations; Total = total number of analyzed sequences.

The Gene Ontology (GO) annotation of the *Citrus *unigene set was performed with BLAST2GO (B2G) [38]. B2G assigns GO annotations through a 3-steps procedure: BLAST against protein databases, retrieval of all GO annotations for a specified number of BLAST hits (Mapping), and GO assignment through an evaluated annotation rule (Annotation). Figure 3A shows the intensity of GO annotation. A total of 10842 unigenes were annotated with 39173 annotations, distributed among the main Gene Ontology categories: Biological Process (11868), Molecular Function (14686) and Cellular Component (12604). There were 5515 sequences annotated with all three GO categories, and 8576 that had at least two annotations. Failure in GO term assignment was due to either a negative result in the BLAST search (NoBLAST, 53%), the absence of GO annotation in any of the BLAST hits (NoMapping, 7%) or because the sequence did not fulfil quality parameters of trustable annotation (NoAnnotation, 40%) (Figure 3B). Noteworthy was that most of these last 47% of sequences, i.e., sequences with a BLAST result without GO term assignment, had best BLAST hits to unknown or hypothetical proteins, indicating the uncertainty in the functional characterization of the hit sequences.

**Figure 4:**
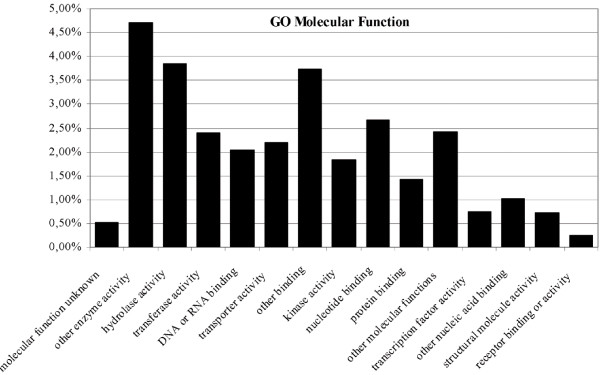
**Slim GO Annotation of the *Citrus *unigene set**. Grey columns indicate the number of Citrus unigenes included in the different slim GO annotation categories.

To provide a general representation of the distribution of *Citrus *gene ontology annotation, the Slim GO Classification for Plants developed at TAIR [33] was obtained, and sets of genes according to broad GO ontology categories were produced [39]. All functional categories in the Biological Processes classification were well represented in the *Citrus *unigene set (Figure 4). Similar results were obtained in the other main GO classes, Molecular function and Cellular Localization (data not shown).

### Characterizing the *Citrus *Gene Space

In an attempt to characterize the *Citrus *gene space, a first analysis was performed to study the biological context of the novel unigenes. Further studies were addressed to identify candidate genes for molecular markers, gene duplications and conserved gene families.

#### Novelty and biological context

The results presented in this work identified more than 5159 sequences that had not been included before in any *Citrus *EST collection, and could be, therefore, novel *Citrus *unigenes. Most of these sequences were derived from the normalized full-length library (4673), that contained a mixed of reproductive and vegetative tissues particularly enriched with fruit tissues, abscission zones and salinity samples. Thus, the unigene set of this library probably included many low abundant transcripts related to several physiological and developmental processes, including fruit quality, productivity and salinity, the targets of this study. Interestingly, 20% of these novel unigenes corresponded to unknown proteins. The number of novel unigenes included into standard libraries was 486, while 148 of them were annotated as unknown genes. To estimate the input of the standard libraries in terms of gene novelty, sets of unigenes known (or assumed) to be involved in the three processes of interest were selected and the libraries containing them identified.

Fruit quality includes many physical attributes and chemical characteristics of the fruit, such as sugar and acid content, flavour and aroma compounds (organoleptic properties). In addition, *Citrus *fruits contain an extensive array of secondary compounds with pivotal nutritional properties. These traits that are acquired along fruit growth are controlled by primary, intermediate, and secondary metabolic pathways. In order to identify genes with relevant roles in fruit quality, homologs of structural enzymes from some of these pivotal metabolic pathways were searched in *Citrus*. The pathways of known topology involved in the biosynthesis of flavonoids and their precursors, the glycolysis, the Krebs cycle, the oxydative/nonoxydative pentose phosphate pathway, the aromatic aminoacid pathway and the general phenyl propanoid and flavonoid pathways were targeted. To identify the *Citrus *unigenes that putatively coded for structural enzymes of the selected pathways, the EC number [40] of the *Citrus *predicted proteins was determined. The results indicated that these pathways were fully represented (Table 4), and that most putative enzymatic activities were present with a significant degree of redundancy, both in terms of EST (expression) and Unigenes (putative paralogs) numbers. From a total of 220 unigenes (1026 ESTs) related to the enzymatic activities of the pathways analyzed in Table 4, it was found that about 15% of them corresponded to novel unigenes. For example, 7 out of the 12 unigenes assigned to the fructose 1,6 biphosphate aldolase activity in the glycolitic pathway are described in this work for the first time. Furthermore, 36% of the 58 enzymatic activities implicated in the processes displayed novel unigenes.

The lignin biosynthesis pathway was analyzed in detail, since lignin is an important component of the dietary fiber present in the fruit [41], that has important benefits for human health [42,43]. This pathway has been recently described in *Arabidopsis thaliana *[44,45], and is also found at the AraCyc database [33]. ESTs with significant similarity to any of the Arabidopsis enzymes involved in this route were selected and reassembled with the Staden Gap4 program [46]. The number of unigenes was estimated by considering only those contigs showing non identical consensus sequences that overlapped significantly. For each enzymatic activity described in the lignin pathway a phylogenetic analysis that included the *A. thaliana *and *Citrus *proteins was carried out. These analyses redefined the number of *Citrus *unigenes that could be considered orthologs of the Arabidopsis genes. The number of *Citrus *ESTs and Unigenes related to the Arabidopsis proteins of the lignin pathway are shown in Table 5. All the enzymatic activities involved in this pathway were represented, and for 8 particular Arabidopsis proteins, *Citrus *possessed 2, 3 or even 5 orthologs according to the phylogenetic analysis A search for enzymes of this pathway performed on the annotation database of the draft sequence of the genome of *Populus trichocarpa *[47], produced similar results, with 22 gene models assigned to the 4-coumarate coenzyme A ligase activity, and 5 gene models to the caffeoyl-CoA O-methyltransferase activity. These data suggest that multiple orthologs of all Arabidopsis lignin proteins could be apparently found if enough number of *Citrus *ESTs were analyzed, indicating the relevance of the lignification process in *Citrus*. The comparison of the unigenes involved in lignin biosynthesis (Table 5) against *Citrus *ESTs from GenBank, showed that 7 out of the 22 orthologs produced not hits, indicating that they were novel genes reported in this work for the first time.

Additional new unigenes implicated in fruit quality were selected based on published information [18,19,48] from relevant pathways of lipids and fatty acid metabolism and degradation [GenBank:DY258371, GenBank:DY258372, GenBank:DY258373, GenBank:DY258374, Contig0424, Contig4859, Contig5406, GenBank:DY258378, GenBank:DY258379, GenBank:DY258380, GenBank:DY258381, Contig0330], synthesis and accumulation of citric acid [GenBank:DY258383, GenBank:DY258384, Contig5931], sugar [GenBank:DY258396] and nitrate transport [Contig3203, Contig4271, GenBank:DY258401, GenBank:DY258402], and chlorophyll synthesis [GenBank:DY258395]. The analyses indicated that 64% of these novel genes were found in the normalized library, while 14 % of them were isolated from FruitTF, one of the two fruit specific libraries. The other unigenes belonged to stress and abscission libraries.

The analysis of genes related to productivity was initially focused in 3 families that had been previously associated with the abscission process, the auxin responsive factors (9 novel unigenes), the receptor protein kinases (35 novel unigenes) and the EREBP (ethylene responsive element binding protein, 4 novel unigenes). The results showed that only one singleton [GenBank:EH405902] of the first family, Contig5401 of the second and Contig5227 of the third one, derived from standard abscission libraries (AbsAOv1, AbsDev, and AbsCOv1), while the remaining members (45), were isolated from the normalized library. Since many of the processes implicated in abscission are controlled by the selective removal of short-lived regulatory proteins, we also analyzed the occurrence of the ubiquitin/26S proteasome pathway [49] among the novel sequences. This component, deeply involved in protein degradation, has not yet been related to abscission and, therefore, no previous information is available in this regard. Interestingly, the only member of E2s Ub-conjugating enzymes [GenBank:DY258370] found in the set of novel unigenes was detected in the AbsDev library. In addition, 4 members out of 23 E3s Ub-ligases [Contigs 5267 and 5546, GenBank:DY257277 and GenBank:DY258093], were exclusively obtained from the abscission-related libraries. These putative unigenes may participate in the removal of repressor elements during the organ separation [23,50,51]. The other Ub-ligases were mostly present in the normalized library. Several cell wall structural proteins [GenBank:DY256701, GenBank:DY257041, GenBank:DY258445 and GenBank:DY258901] and two specific glycosyl hydrolases [GenBank:DY257803 and GenBank:DY258004] were exclusive of the abscission-related libraries, suggesting that abscission may also implicate active remodelling of cell walls [52]. Aside from the normalized library, the abscission libraries that were strongly enriched with specific abscission zone tissues, showed a relatively high number of novel exclusive unigenes (162).

For the analyses of novel citrus genes potentially involved in abiotic stress, the following well established biological functions were investigated: sodium Na+/H+ antiporters [GenBank:DY258370, GenBank:DY305688 and GenBank:DY300954], that are probably involved in sodium detoxification [53]; the Calcineurin B gene homolog (Contig 5589) [54], stress-induced and/or -activated protein kinases [GenBank:DY278709, Contig0907, Contig1053 and GenBank:DY291464] and the mechano-sensitive ion channel-domain containing protein [GenBank:DY262334], that are likely implicated in NaCl-associated signal transduction mechanisms; aldehyde dehydrogenases involved in detoxification [GenBank:DY301300 ] [55]; two genes of the inositol metabolism [GenBank:DY304982, GenBank:DY260177, GenBank:DY261021, GenBank:DY270505]; genes associated with lipid metabolism such as the phosphoinositide-specific phospholipase C [GenBank:DY260755] and the lipoxygenases [GenBank:DY258546 and Contig5406]; and the membrane-associated salt-inducible protein [GenBank:DY301464]. Moreover, the following biological functions related to acclimatization to osmotic shock were searched: two different NCED4 genes involved in ABA biosynthesis (Contig0189 and Contig0309) [24]; and two genes involved in trehalose metabolism [GenBank:DY280731 and GenBank:DY294040]. Lastly, cell tolerance mechanisms universally linked to different abiotic stresses, represented by heat shock proteins and molecular chaperons [GenBank:DY261699, GenBank:DY258174, GenBank:DY303256, GenBank:DY270445, GenBank:DY258682, GenBank:DY258174, GenBank:DY271306, GenBank:DY303256, GenBank:DY270445, GenBank:DY257994, GenBank:DY260682][56]; and uncharacterized stress-responsive genes [Contig0907, Contig2771, GenBank:DY270558 and GenBank:DY260600] were also analyzed. The results of this search indicated that 42% of these abiotic stress related genes were detected in the normalized library, while 34 % of them was found in fruit libraries (FruitTF and PhII-IIIVesicles1). Only 1 unigene, a putative sodium Na+/H+ antiporter [GenBank:DY305688], was found as a singleton in a salinity-related library, LSH, whereas only Contig5589 (Calcineurin B gene) contained ESTs exclusively derived from KCl-Salt1, another salinity library.

Overall, these preliminary estimates showed that most of the novel genes, presumably implicated in fruit quality, abscission, and salinity responses, were effectively recovered in the normalized library. The contribution of the fruit and abscission libraries to the set of unigenes related to fruit quality or abscission, could be roughly estimated between 5 and 15%. The lower contribution of the salinity standard libraries (less than 5%) may be due to the abundance of unspecific cross-responses among multiple abiotic and biotic stresses. For an accurate estimation of these figures, however, confirmation of gene specificity appears to be mandatory.

#### Molecular markers

The use of genetic and molecular markers is crucial to facilitate the identification and cloning of genes of agronomic interest [57], while single copy genes are usually good candidates to be used as markers. To identify conserved *A. thaliana *orthologs present as single copy genes in both Arabidopsis and *Citrus*, a database with 3700 Arabidopsis single copy genes was obtained from the Compositae Genome Project Database [58], and used in a BLAST search with the *Citrus *unigene set as queries. A total of 726 *Citrus *sequences showed an unambiguous single strong BLAST hit, and reciprocal BLAST searches (Arabidopsis single copy genes versus the EST assemblies) produced the same results. The outcome of this BLAST search was compared with that obtained in the BLAST search performed against all *Citrus *ESTs from GenBank. The results showed that 129 unigenes did not generate any hit, while 445 clusters only produced hits with similarities higher than 95%, suggesting that these were ESTs probably derived from the same transcript. Although this analysis is not conclusive, the absence of hits or the occurrence of extremely high similarities, suggested that these 574 unigenes are strong candidates to be conserved orthologs of Arabidopsis single copy genes.

#### Gene duplications

The BLAST search with the Arabidopsis single copy genes also produced 234 sequences with 2 or more strong *Citrus *hits. These cases were further investigated as they might be indicative of gene duplication events produced in the *Citrus *genome. In many cases, Unigenes showing the same Arabidopsis hit did not overlap, indicating that they may derive from the same transcript but were not assembled in the same contig, and therefore cannot be considered to be different genes. Finally, 18 Arabidopsis single copy genes showed strong similarity with two overlapping *Citrus *unigenes. These clusters presented the same Arabidopsis protein as their best hit, supporting the hypothesis that they are paralog genes in *Citrus *[see Additional file 1].

#### Gene Family analysis

Comparative genomics was used to characterize the conserved gene families in *A. thaliana *and *Citrus *species. There are currently 930 gene families, comprising 6399 genes, described at the *Arabidopsis thaliana *Information Resource database [59]. The presence of these gene families in the *Citrus *unigene set was explored, allocating the *Citrus *clusters in the gene families based on the best Arabidopsis significant hit obtained. About 3000 *Citrus *unigenes were assigned to 724 families, and 52 super families, showing that 78% and 92% of the Arabidopsis families and superfamilies were represented in the *Citrus *EST collection. To exemplify the potential for *Citrus *improvement of the information included in the EST collection, two gene families with relevant agronomic interest were selected and analyzed in detail: the ammonium transporter family intimately related to plant nutritional efficiency and the glycoside hydrolase family 20, implicated in sugar synthesis in fruit.

**Figure 5:**
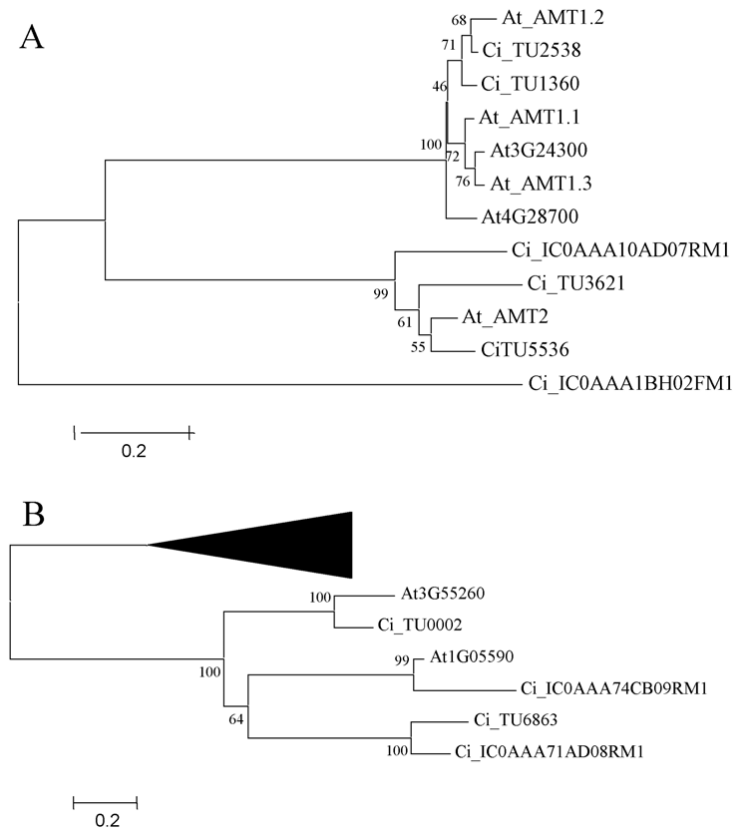
**Phylogenetic analysis**. A – Phylogenetic tree of the ammonium transporter family from *A. thaliana *and *Citrus *species. B – Phylogenetic tree of the glycosyltransferase family 20 from *A. thaliana *and *Citrus *species. Glycosyltransferase family 19 cluster was collapsed in a black triangle and used as an outside group.

The Arabidopis high-affinity ammonium transporter family is composed of six members: five proteins that form the AtAMT1 subfamily [60] and a member, AtAMT2, that is distantly related to the AtAMT1 subfamily [61]. Similarity searches showed that 60 *Citrus *ESTS were significantly similar to the Arabidopsis ammonium transporters. These ESTs that corresponded to 6 unigenes (4 contigs and 2 singletons) were translated into protein with prot4EST. A multiple alignment of the *A. thaliana *and *Citrus *sequences was performed with Clustal X, and a phylogenetic tree was constructed with the neighbor joining method (Figure 5A). The tree showed that 3 *Citrus *unigenes closely related to AtAMT2 grouped together in a cluster supported by very high bootstrap values. Thus, the AMT2 subfamily of ammonium transporters in *Citrus *comprised, at least, 3 proteins, suggesting the occurrence of a number of duplication events.

Glycosyltransferase family 20 is composed of proteins with known α, α-trehalose-phosphate synthase UDP-forming activity, and in *A. thaliana *comprises proteins At1G05590 and At3G55260. A total of 15 *Citrus *ESTs displaying significant similarity with the glycosyltransferase family 20 proteins were assembled into 4 unigenes grouped in 2 contigs and 2 singletons. As above, phylogenetic analysis showed that the *Citrus *and Arabidopsis proteins clustered together, with high bootstrap values supporting the clade (Figure 5B). This analysis suggests that the glycosyltransferase family 20 included 4 members in *Citrus *species while in *A. thaliana *it contained only two proteins.

## Discussion

Citrus is the main fruit tree crop in the world. However, traditional breeding for *Citrus *cultivar improvement faces many serious impediments due to the unusual combination of biological characteristics of *Citrus*, their low genetic diversity and the long-term nature of tree breeding. Genomic technology can overcome these limitations providing new tools, for example, to produce more efficient varieties and rootstocks and to identify new genes, alleles or genotypes of agronomic relevance. Improvement of knowledge of the transcriptome is one of the first tasks that have to be developed in order to understand the developmental biology of the plants and how these respond to environmental stresses. This work that pursues this goal provides a deep insight into the *Citrus *transcriptome specifically related to three major commercial traits i.e improved fruit quality, higher yield and tolerance to environmental stresses, especially salinity.

Towards this objective, 10 cDNA libraries representing particular treatments and tissues from selected varieties and rootstocks differing in fruit quality, resistance to abscission and tolerance to salinity were generated to provide a large and enriched expressed sequence tag collection. The assembly of these sequences, more than 52600 ESTs, allowed the identification of 15660 transcription units. The results of this analysis are comparable to previous reports in *Solanum tuberosum*, that detected 19892 unigenes from 61949 ESTs [62], or in *Sorghum bicolor*, with 16801 unique transcripts derived from 55783 ESTs [63]. The data showed that all sequences from the *Citrus *species analyzed, from both this study and databases, were almost identical, suggesting that the differential behaviour of these cultivars during normal fruit growth or when facing environmental adverse conditions is more likely associated with differences in gene regulation rather than with sequence divergence. This result is not unexpected since *Citrus *posses a high level of phenotypic diversity while global genetic diversity, analyzed with molecular markers, appears to be very low or practically null. A large effort was made to determine the real number of different transcripts represented in the EST collection. It is well known that the accuracy of EST clustering is affected by various error sources, such as sequencing mistakes, contaminant sequences and the presence of products of chimeric splicing. The most common error occurs when different ESTs from the same gene are falsely separated into two or more clusters [64]. To overcome this difficulty the level of redundancy was estimated comparing all unigenes with each other, and clustering them in *supercontigs*, that are more likely to correspond to real transcripts. The level of redundancy was estimated to be 26%, a value similar to that obtained in sugarcane, for example [65]. This first restriction suggests that the likely number of unigenes in the *Citrus *collection is closer to 13900 rather than to 15660.

It was also crucial to determine the occurrence of contaminant sequences, mostly from microorganisms, since many samples were taken from open field. The presence of contaminant sequences (mainly from bacteria and fungi) is a general problem not attended in any of the EST projects we have examined. For instances, a BLASTN search performed with several contaminant sequences found in this work against the viridiplantae section of the GenBank EST database revealed a considerable number of ESTs regarded as plant sequences that really corresponded to fungi species (data not shown). Thus, the analysis reported in this work may help to prevent the presence of sequences from contaminant species in the databases. Determining the species of the unigenes best hit sequences helped to identify putative contaminants, allowing not only a more precise estimation of the real number of *Citrus *transcripts but also criteria for microarray EST selection. Since about 400 Unigenes were believed to be contaminant sequences from other species, the number of *Citrus *expressed genes was reduced to 13500.

A relevant observation of this work is that more than 38% of the 13500 unigenes (5159 sequences) are novel *Citrus *unigenes. EST sequences were obtained from two kinds of cDNA libraries, normalized full length and standard libraries. The normalized library was generated with a wide variety of reproductive and vegetative tissues, enriched with developing fruits, abscission zones and salinity samples. In the first strategy, the normalization process very effectively increased low abundant transcripts, since the bulk of the novel unigenes described (4673) derived from this library. The standard libraries that were constructed from either samples of fruits, abscission zones or salt-treated organs, were generated with the idea of providing transcripts specifically expressed at these particular tissues and organs without increasing redundancy. To estimate the contribution of the standard libraries in terms of gene novelty, a set of unigenes presumably involved in the three processes of interest was selected and the libraries containing them identified. Although for an accurate evaluation of these contributions confirmation of tissue specificity appears to be mandatory, these preliminary estimates showed that most of the novel genes were certainly recovered in the normalized library. Moreover, the contribution of the specific standard libraries to the unigene set maybe roughly estimated to be between 4 and 15%.

The primary homology searches performed against different databases allowed annotation of most unigenes, with more than 73% of them displaying a similarity degree higher than 60%. These results agreed those obtained in previous *Citrus *[12] or sugarcane [65] EST projects. It was also shown that most of the sequences that did not produce significant hits in the BLASTX searches were shorter than 500 bp (Fig 2) and probably did not carry coding sequences. Additional efforts were performed to characterize these sequences, with supplementary BLASTN searches against the non redundant and EST nucleotide databases. These analyses gave rise to the suggestion that 647 ESTs of the *Citrus *unigene set may correspond to *Citrus *exclusive genes since the significant hits they produced were only for *Citrus *sequences, in spite of the more than 8.5 million EST sequences derived from plant species deposited at the GenBank,

Further improvement of the annotation was carried out through searches performed against secondary databases, composed of patterns or signatures. Although these prediction methods can work with DNA sequences, the error prone nature of ESTs, mainly shifts in the reading frame (missing or inserted bases) or ambiguous bases, may result in inaccuracies and loss of information. Thus, a crucial step in annotation is the robust translation of the ESTs to yield predicted polypeptides. Polypeptide sequences posses a better template for almost all annotation tools, including InterPro and Pfam, and allow the assembly of more accurate multiple sequence alignments. High quality polypeptide predictions can be applied to functional annotation and post-genomic study in a similar way to those available for completed genomes. In the work presented here, the protein translation was performed with Prot4EST, a prediction pipeline that incorporates freely available software (ESTscan, Decoder, HSP tiling) to produce final translations that are more accurate than those derived from any single method [36]. The use of the interProScan tool [37], allowed simultaneous search for motifs against 9 databases. This search produced significant results for almost 11000 predicted proteins, including 342 unigenes that did not have significant hits in the BLAST searches. From the 20 most abundant motifs found in *Citrus *with Pfam, 7 of them also were included in the top 20 list at the Pfam database, enforcing the accuracy of the analysis and the representativity of the *Citrus *EST collection. The molecular functions associated with these 20 motifs can be grouped in 4 categories: protein-protein interaction (47.26%), nucleic acid binding (27.51%), protein modification and binding (14.9%), and calcium metabolism (6.17%), which indicates the relative significance of these cellular functions in *Citrus*.

The distribution of motifs on the polypeptide sequences predicted to be complete proteins showed that the bulk of sequences displayed a single motif (76%). Proteins carrying 2 ore more motifs showed unlike signatures rather than repeats of the same motif. For instance, the number of proteins with 2 different signatures (452) was six times higher than the number of proteins that had 2 identical motifs (69). Similar relationships were found for other number of repeats.

The Gene Ontology annotation of the *Citrus *unigenes was performed with BLAST2GO (B2G), a recently developed BLAST-based GO annotation software [38]. The B2G approach uses multiple BLAST hits to search for functional annotations and assigns GO terms to the query sequence applying an annotation algorithm that considers HSP length, e-value, percentage of similarity, Evidence Code of the source annotations and the topology of the Gene Ontology. This is in contrast to most EST projects that perform annotation solely by direct assignment of the GO terms to the best hit of BLAST searches [12,66,67]. The B2G method has shown to have a high annotation recall and has been used in other EST projects in eukaryotes [68].

Metabolic pathways responsible of important agronomic traits were further surveyed to determine the extent of representation of these pathways within the *Citrus *unigene set. In addition to the finding that most enzymatic steps were represented by *Citrus *homologs, a preliminary estimate of gene duplication based on the occurrence of paralogous sequences was also provided. Defining such relationships and understanding functional diversification of paralogs is an important field of research in genomics-assisted crop improvement. Lignin biosynthetic pathway was the object of a deeper analysis since *Citrus *fruits are very rich in products with beneficial effects in preventing cancer, diabetes, ...etc such as fiber [42,69]. Dietary fiber, that consists of non digested structural and storage polysaccharides and lignin, lowers cholesterol levels and helps to normalize blood glucose and insulin levels [70]. The detailed analysis of lignin biosynthesis pathway, carried out in *Citrus*, indicated that in comparison with Arabidopis, *Citrus *possessed at least 9 additional enzymatic activities involved in lignin synthesis (Table 5). Furthermore, the results obtained for gene models from *Populus trichocarpa *[47], also appears to support the idea that the extensive formation of secondary xylem in tree species, requiring high levels of lignin synthesis may have been the origin of the expansion of the genes involved in this pathway.

More than 570 unigenes have been suggested to be possible conserved orthologs of Arabidopsis single copy genes. Recent studies have indicated that ancient polyploidy is common across angiosperm lineages and in fact, the genomes of all angiosperms may have been influenced by at least one genome-wide duplication event [71]. Despite such events, single-copy, apparently orthologous gene sets have been identified in a broad range of angiosperms [72,73]. Selection against duplicates may be maintaining these genes as single copy, and therefore are precious markers for comparative genetic and physical mapping, and also for phylogenetic analyses [74]. Identification of such genes in *Citrus *species is mandatory to perform this kind of analyses [75]. The study also revealed a number of genes that might be duplicated in the *Citrus *genome, while remained as single copy genes in Arabidopsis, although the possibility of finding additional copies of these genes could not be discarded, when the whole transcriptome of *Citrus *is available. If these duplications are the result of individual events or were caused by a genome-wide duplication cannot be answered with the current information.

Comparative genomics was also used to obtain an overview of conserved gene families in *Citrus*. All Arabidopsis gene families studied were well represented in the *Citrus *EST collection, although the number of their members was generally smaller, probably because the unigene set was only a partial representation. For the same reason, the finding of families that in *Citrus *clearly outnumbered their Arabidopsis counterparts is highly significant. The phylogenomic analysis performed on the gene families of ammonium transporters and glycosyltransferases supported this idea confirming the occurrence of additional members in the *Citrus *families. Ammonium is one of the prevalent nitrogen sources for growth and development of higher plants including *Citrus*. The ammonium transporter family is composed in Arabidopsis of 5 AMT1 related genes and AMT2, which is more closely related to ammonium transporters from prokaryotes than to AMT1. AtAMT2 is likely to play a significant role in moving ammonium between the apoplast and symplast of cells throughout the plant [61]. Interestingly, there are three AMT2 like genes in *Citrus *(Fig 6A). Glycosyltransferases are a ubiquitous group of enzymes that catalyse the transfer of a sugar moiety from an activated sugar donor onto saccharide or non-saccharide acceptors. Although many glycosyltransferases catalyse chemically similar reactions, they display remarkable diversity in their donor, acceptor and product specificity and thereby generate a potentially infinite number of glycoconjugates, oligo- and polysaccharides. [76]. Thus, the additional members found in this family, might be related to the complexity of sugar synthesis that takes place in the *Citrus *fruits.

## Conclusion

The assembly of more than 54000 *Citrus *ESTs from five cultivars differing in basic fruit developmental aspects, such as major traits for fruit quality and production, and in the responses to environmental conditions, provides an unprecedented insight of the *Citrus *transcriptome. This study contributes new tools for *Citrus *genetic and genomic analyses. The unigene set, composed of ~13000 putative different transcripts, including more than 5000 novel Citrus genes, was assigned with putative functions based on similarity, GO annotations and protein domains. In addition, comparative genomics was used to analyze the *Citrus *transcriptome, and evidences for numerous cases of gene duplication events were presented. The similarity analyses indicated that the sequences of the genes belonging to the varieties and rootstocks studied were essentially identical suggesting that the differential behaviour of these species cannot be attributed to main sequence divergences. This set of processed EST sequences has greatly contributed to the development of a new *Citrus *microarray.

## Methods

### 1. Plant material

The *Citrus *genotypes used to generate the cDNA libraries were the varieties *Citrus clementina*, (cv Clementina de Nules), and C. *sinensis *(cvs Navelina and Washington Navel), and the rootstocks *C. reshni *(cv *Cleopatra *mandarin) and *C. sinenis *× *Poncirus trifoliata *(cv Carrizo citrange). Their characteristics are as follows. Clementine is a mandarin of elevated fruit quality, high ovary and fruitlet abscission and moderate salt tolerance. Washington Navel is a late sweet orange that generally shows pre-harvest abscission. In contrast, Navelina, an early orange variety, exhibits low fruit abscission but higher salt sensitivity. *Cleopatra *mandarin is an efficient salt tolerant rootstock while the hybrid Carrizo citrange shows high salt sensitive).

### 2. Normalized Full Length Library (NFL)

#### Tissue Samples and Treatments Description

All samples included in the normalized full-length library were harvested from *Citrus clementina *(cv Clementina de Nules). They were composed of the following tissues and organs: developing vegetative tips and buds, dormant buds, developing leaves, shoots, internodes and roots, abscission zones from leaves, flowers, ovaries and fruits, flowers and inflorescences, growing and senescent ovaries, developing fruitlets (stages I & II), flavedo from growing, ripening and, senescent fruits and fruit flesh (juice sacs, stages I, II & III). The library also included leaves subjected to different treatments: short- and long-term salinity, drought and rehydration, mineral deficiencies, alkaline and calcareous soils, low and high temperature, flooding, oxidative stress, wounding, insect attacks, and elicitors (harpin) treatments. All tissues were frozen in liquid nitrogen and equal amounts of homogenized tissues were mixed in a single sample for total RNA extraction.

#### Library Construction

Full-length cDNA synthesis was carried out with Invitrogen proprietary RNase H reduction reverse transcriptase "cocktail" for mRNA isolation, 5' cap full-length enrichment, and the reduction of oligo(dT)-priming. Normalization was carried out by self-subtraction, with Invitrogen technology, as described by manufacturer. Normalization produced a 24 fold average reduction of the abundant clones, (from 0.16% abundance to 0.0065%). PCMVSport6.1 was used as a cloning vector.

### 3. Standard Libraries

#### Tissue Samples and Treatments Description

- **Fruit-TF**: parthenocarpic fruits of *Citrus clementina *(cv Clementina de Nules) mandarin were harvested from adult trees grown grafted onto Carrizo citrange rootstock (*Citrus sinensis *× *Poncirus trifoliata*) in a homogeneous orchard under normal culture practices. Flavedo (exocarp) samples were isolated from fruits collected on July 28 (69 days post anthesis, dpa), July 24 (85 dpa), August 2 (94 dpa), October 11 (164 dpa), November 18 (202 dpa), November 25 (209 dpa), December 13 (227 dpa) and January 9 (254 dpa). Samples of fruit flesh, consisting of juice vesicles (endocarp) including the segments with their membranes and vascular bundles, were taken from fruits collected on May 13 (13 dpa) and June 10 (41 dpa). Samples were frozen under liquid nitrogen and stored at -80 \circC until RNA isolation. Mixtures of equal amounts of poly-A+ RNA from the samples were used.

- **PhII-IIIVesicles1**: fruit juice vesicles from Clementine *g*rafted onto Carrizo citrange were taken at one month intervals: July 8 (69 dpa), August 2 (94 dpa), September 12 (135 dpa), October 16 (169 dpa), November 14 (198 dpa) and December 17 (231 dpa). A mixture of equal amounts of poly-A+ RNA from the six samples was used.

- **AbsDev**: laminar abscission zone and surrounding tissues (petiole and blade) of developing leaves were harvested from Clementine on Carrizo citrange.

- **AbsCFruit1**: abscission zone C and surrounding tissues of ripe fruits were harvested from *Citrus sinensis *(cv. Washington navel) scions on Carrizo.

- **AbsCOv**: abscission zone C and surrounding tissues of ethylene-treated ovary explants were harvested from Clementinescions on Carrizo.

- **AbsAOv1**: abscission zone A and surrounding tissues of ethylene-treated ovary explants at "petal fall" stage were harvested from Clementine scions on Carrizo.

- **LSH**: leaves were harvested from one-year-old *Citrus sinensis *(cv Navelina) scions grafted onto Cleopatra (*Citrus reshni*) rootstock cultured under salinity conditions. Potted plants were grown in greenhouse conditions and subjected to regular irrigations (three times per week) with 25 mM NaCl:CaCl2 solutions for 60 days.

- **KCl-Salt1**: non-suberized roots, enriched in distal (actively growing) root portions were harvested from 1 year-old Cleopatra mandarin seedlings. Potted plants grown in greenhouse conditions were subjected to Cl-starvation and resupply treatments at different times with 50 and 100 mM KCl.

- **EHR**: young roots from Carrizo citrange were collected 3, 6, 12, and 24 hours after water stress treatment and 1, 6, and 10 hours after re-watering. A mixture of equal amounts of poly-A+ RNA from the different samples was used.

#### RNA Extraction

For AbsDev, AbsCFRuit1, AbsCOv1, AbsAOv1, and EHR libraries, total RNA was isolated from frozen tissue using the standard guanidine protocol [77]. For FruitTF library, total RNA was isolated from frozen tissue using the RNeasy Plant Mini Kit (Qiagen) and treated with RNase-free DNase (Qiagen) through column purification according to the manufacturer's instructions. For KCL-Salt1 library, total RNA was isolated from frozen tissue using acid phenol extraction and Lithium Chloride precipitation method [78]. In all cases, RNA quality was assessed by espectrophotometry and gel electrophoresis [77].

#### Poly(A)+ RNA Isolation

Poly(A)+RNA was isolated from a mixture of equal amounts of total RNA from all samples using the Oligotex mRNA Mini Kit (Qiagen) following manufacturer's instructions.

#### Construction protocols and cloning vectors

KCl-Salt1, AbsDev, AbsCFruit1, and FruitTF cDNA libraries were constructed using the CloneMiner cDNA Library Construction Kit (Invitrogen) with the pDONRTM222 vector. AbsCOv1, AbsAOv1, and EHR cDNA libraries were constructed with SMART cDNA Library Construction Kit (Clontech) and pTriplEx2 as the cloning vector. SLH library was constructed with Stratagene cDNA synthesis kit and the pBluescript SK (-) vector. PhII-III-Vesicles1, and EHR libraries were constructed using the UNI-ZAP XR and Gigapack III Gold kits from Stratagene and ë-ZAP II cloning vector.

### 4. EST assembly and annotation

DNA templates were prepared using the 96-well alkaline lysis DNA method. Sequencing was performed using the ABI Big Dye Terminator Cycle Sequence Ready Reaction as described by manufacturer, with the T7 forward primer in 96 well plates in an automatic ABI 3730.

The software phred was used for base calling, and Crossmatch for vector masking [79]. Reading assembly was performed with the CAP3 program [30], using read quality and defaults parameters. Similarity searches were performed with the standalone version of BLAST [31], against the NCBI non redundant protein, nucleotide and EST databases [32], the *Arabidopsis thaliana *protein set from TAIR database [33] and the *Oryza sativa *protein set from TIGR Rice database [80]. Parsing of the BLAST results was performed with the Bio::SearchIO module [81] from the Bioperl package [82].

Protein translation was performed with prot4EST polypeptide prediction pipeline [36], which combines different methods like ESTscan [83], DECODER [84] and similarity search results (BLASTX) to produce accurate translations. Motif search was performed with the standalone version of the interProScan tool that combines the protein function recognition methods of the database members of InterPro into one single application [37]. The InterPro database unites the following secondary databases: Uniprot [85], Panther [86], PROSITE [87], PRINTS [88], Pfam [89], ProDom [90], SMART [91], TIGRFAMs [92], PIR [93] and SUPERFAMILY [94].

Gene Ontology annotation of unigenes was performed with BLAST2GO [38]. Blast2GO is a user adjustable tool that utilizes BLAST to find homologous sequences for a set of query sequences and returns an evaluated annotation from the gene ontology annotations present in the BLAST hits of each sequence. B2G parameters were: NCBI non-redundant DB for BLAST search, 20 hits maximum for BLAST result, 100 nt as minimum HSP-length to retain putative annotating hits and default Evidence Code Weights for Gene Ontology annotation that assigns high ECWs to experimental-based and curated annotations while penalized electronic and non-curated annotations. Minimum values for BLAST e-value and % similarity of the BLAST result were e-06 and 55% respectively and ultimate annotation cut-off value was set to 55. This set of parameters was shown to provide the most reliable results in the annotation of Arabidopsis sequences [38]. GOSlim annotations of the *Citrus *unigenes were also generated with the B2G software using the plant GOSlim mapping provided in TAIR.

### 5. Gene space analysis

Single copy gene set from *A. thaliana *was obtained from the Compositae Genome Project Database [58] and used as query in a BLAST search against a database generated with the *Citrus *unigenes. These results were compared with those obtained with the BLASTX search performed with the *Citrus *unigenes against the Arabidopsis complete protein set. Only Unigenes with a unique Arabidopsis significant hit that matched the results obtained with the first BLAST search were considered to be putative orthologs of the Arabidopsis single copy genes. A similar approach was used to detect possible gene duplications, selecting those *Citrus *unigenes that had as significant hits the same *A. thaliana *protein. ESTs corresponding to the selected unigenes were reassembled with the Staden package [46] to confirm that the Unigenes were overlapping rather than identical sequences.

The *A. thaliana *gene family dataset was obtained from TAIR [33] and the Arabidopsis best hit of the *Citrus *unigenes was used to find *Citrus *representatives of relevant families. ESTs from the overrepresented *Citrus *families were selected and reassembled with the Staden package [46], and only overlapping non-identical consensus sequences were considered for further analysis. For the phylogenetic study of the ammonium transporter and glycosyltransferase 20 families, a multiple proteins sequence alignment was carried out with ClustalX [95], genetic distances were calculated with the protein correction for the poisson method [96], and phylogenetic trees were constructed with the neighbor joining tree method [97], using the Molecular Evolutionary Genetic Analysis software package MEGA3 [98].

## List of abbreviations

aa, amino acid

bp, base pair

EST, expressed sequence tag

GO, gene ontology

nt, nucleotide

TAIR, The Arabidopsis Information Resource

TIGR, The Institute for Genomic Research

## Authors' contributions

JT carried out bioinformatics (assembly, annotation and phylogenetic analyses) and drafted the manuscript. AC participated in the GO annotation of the sequences and helped to draft the manuscript. MC participated in sample collection and construction of cDNA libraries. JMC participated in sample collection, construction of cDNA libraries, and helped to draft the manuscript. FT participated in sample collection and construction of cDNA libraries. JA participated in sample collection and construction of cDNA libraries. EA participated in sample collection and construction of cDNA libraries. FA participated in sample collection and construction of cDNA libraries. XA contributed to the annotation of the sequences. JB participated in sample collection and construction of cDNA libraries. GS participated in sample collection and construction of cDNA libraries. BC performed EST sequencing. CD performed EST sequencing. SG participated in the GO annotation of the sequences. DJI generated samples for library construction. FL generated samples for library construction. RM contributed to the coordination and design of the project. PO contributed to the coordination and design of the project. PW performed EST sequencing. MT conceived the study, coordinated the project and drafted the manuscript. All authors read and approved the final manuscript.

## Supplementary Material

Additional file 1 Putative gene duplications in Citrus. The table contains the *A. thaliana *loci that have been foun duplicated in *Citrus*. Click here for file

## Figures and Tables

**Table 1:** Summary of *Citrus *EST libraries

**Species**	**Library**	**Library description**	**Type**	**ESTs**^a^	**Exclusive unigenes**^b^
*C. clementina*	AbsAOv1	Abscission zone A and surrounding tissues of ethylene-treated ovary explants	Standard	900	229
	AbsCOv1	Abscission zone C and surrounding tissues of ethylene-treated ovary explants	Standard	947	240
	AbsDev	Laminar abscission zone and surrounding tissues (petiole and blade) of developing leaves	Standard	877	258
	FruitTF	Flavedo and juice vesicles from fruits at different developmental stages	Standard	3999	1263
	PhII-IIIVesicles1	Juice vesicles from fruits at phases II and III	Standard	1020	70
	NFL	Organs and tissues at different stages. Plants under normal culture practices or subjected to biotic and abiotic stresses	Normalized Full length	41288	8440
*C. sinensis*	AbsCFruit1	Abscission zone C and surrounding tissues of mature fruits	Standard	783	218
	LSH	Leaves from prolonged salt-treated plants	Standard	1009	340
*C. reshni*	KCl-Salt1	Roots subjected salinity (Cl-) treatments	Standard	960	253
*Citrus sinensis *× *Poncirus trifoliata*	EHR	Roots subjected to water stress and re-hydration	Standard	849	121
			**Total**	52632	11432

^a ^Total number of ESTs assembled

^b ^Unigenes (singletons and contigs) from a single library

**Table 2:** The 20 most abundant conserved motifs found with Pfam

Pfam Motif Description	Pfam Motif	N°^a^	Molecular Function ^b^
Leucine Rich Repeat	PF00560.19	457	protein-protein interaction
WD domain, G-beta repeat	PF00400.18	341	protein-protein interaction
PPR repeat	PF01535.9	218	RNA binding
RNA recognition motif. (a.k.a. RRM, RBD, or RNP domain)	PF00076.10	190	nucleic acid binding(GO:0003676)^c^
Protein kinase domain	PF00069.13	156	protein kinase activity (GO:0004672)^c ^ATP binding (GO:0005524)^c^
Ankyrin repeat	PF00023.16	149	protein-protein interaction
EF hand	PF00036.18	111	calcium ion binding (GO:0005509)^c^
Mitochondrial carrier protein	PF00153.13	97	binding (GO:0005488)^c^
Myb-like DNA-binding domain	PF00249.17	87	DNA binding (GO:0003677)^c^
F-box domain	PF00646.19	79	protein-protein interaction
Tetratricopeptide repeat	PF00515.14	77	protein-protein interaction
Armadillo/beta-catenin-like repeat	PF00514.10	77	protein-protein interaction
Zinc finger, C3HC4 type (RING finger)	PF00097.11	74	ubiquitin-protein ligase (GO:0004842)^c ^zinc ion binding (GO:0008270)^c^
Zinc knuckle	PF00098.10	67	nucleic acid binding(GO:0003676)^c^
Zinc finger C-x8-C-x5-C-x3-H type	PF00642.13	67	nucleic acid binding(GO:0003676)
AP2 domain	PF00847.9	58	transcription factor (GO:0003700)^c^
XYPPX repeat	PF02162.6	55	unknown
Kelch motif	PF01344.13	49	unknown
Ubiquitin family	PF00240.12	45	protein modification (GO:0006464)^c^
IQ calmodulin-binding motif	PF00612.14	43	calmodulin binding

^a ^Number of motif repeats found in the Citrus protein set.

^b ^Molecular function associated with the functional domain.

^c ^Molecular function according to the Gene Ontology annotation system.

**Table 3:** Distribution of PFAM motifs in the hypothetical complete proteins

Different motifs per protein^a^	Motif number per protein^b^	Protein number^b^	%	Total Protein number (%)^d^
1	1	1766	71.1%	1890 (76.15%)
	2	69	2.78%	
	3	13	0.52%	
	4	21	0.85%	
	5	7	0.28%	
	6	8	0.32%	
	7	4	0.16%	
	9	2	0.08%	

2	2	452	18.2%	507 (20.43%)
	3	25	1.01%	
	4	18	0.73%	
	5	2	0.08%	
	6	5	0.20%	
	8	5	0.20%	

3	20	1	0.04%	73 (2.94%)
	3	64	2.58%	
	4	5	0.20%	
	5	1	0.04%	
	6	2	0.08%	

4	4	12	0.48%	12 (0.48%)

^a ^Number of different conserved motifs found in a protein

^b ^Total number of motifs found in a protein

^c ^Number of proteins displaying the number of conserved motifs shown in the 2^nd ^column

^d ^Number of proteins displaying the number of different conserved motifs shown in the 1^st ^column

**Table 4:** Primary, Intermediate, and Secondary Metabolic Pathways in Citrus

**Pathway**	**Enzymatic Activity**	**EC reaction**	**Unigenes**^a^	**ESTs**^b^
**Glycolysis**	Hexokinase	2.7.1.1	4 (2)^c^	6 (4)^d^
	Glucose-6-phosphoisomerase	5.3.1.9	6 (1)	16 (1)
	6-Phosphofructokinase	2.7.1.11	10	35
	Fructose 1,6-biphosphate aldolase	4.1.2.13	12 (7)	71 (9)
	Triose phosphate isomerase	5.3.1.1	8 (1)	22 (1)
	Glyceraldehyde 3-P dehydrogenase	1.2.1.12	8 (2)	52 (8)
	Phosphoglycerate kinase	2.7.2.3	6 (1)	15 (3)
	Phosphoglycerate mutase	5.4.2.1	1	5
	Enolase	4.2.1.11	7 (4)	28 (5)
	Pyruvate kinase	2.7.1.40	10 (1)	69 (9)

**Tricarboxylic acid cycle (Krebs cycle)**	Citrate synthase	4.1.3.7	2	13
	Aconitase	4.2.1.3	6 (1)	31 (1)
	Isocitrate dehydrogenase	1.1.1.42	3	59
	a-Ketoglutarate dehydrogenase complex	1.2.4.2	No hits	No hits
	Succinyl-CoA synthetase	6.2.1.5	2	14
	Succinate dehydrogenase	1.3.5.1	2	20
	Fumarase	4.2.1.2	3 (2)	30 (5)
	Malate dehydrogenase	1.1.99.16	4	24

**Oxidative/nonoxidative pentose phosphate pathway**	Glucose 6-P-1-dehydrogenase	1.1.1.49	4	4
	6-Phosphogluconolactonase	3.1.1.31	2	10
	6-Phosphogluconate dehydrogenase	1.1.1.44	15	47
	Ribose-5-P isomerase	5.3.1.6	3	12
	Ribose-5-P 3-epimerase	5.1.3.1	No hits	No hits
	Transketolase	2.2.1.1	3 (2)	22 (11)
	Transaldolase	2.2.1.2	4 (2)	20 (17)

**Aromatic amino acid biosynthesis**	3-Deoxy-D-arabino-heptulosonate 7-P synthase	4.1.2.15	No hits	No hits
	3-Dehydroquinate synthase	4.2.3.4	2	2
	3-Dehydroquinate dehydratase	4.2.1.10	2	2
	Shikimate 5 dehydrogenase	1.1.1.25	1	14
	Shikimate kinase	2.7.1.71	7	10
	5-Enolpyruvoylshikimate 3-P synthase	2.5.1.19	No hits	No hits
	Chorismate synthase	4.2.3.5	2	2
	Chorismate mutase	5.4.99.5	1	1
	Prephenate dehydratase	4.2.1.51	3	5
	Prephenate dehydrogenase	1.3.1.12	2 (1)	18 (2)
	Aromatic amino acid transaminase	2.6.1.57	6	18
	Anthranilate synthase	4.1.3.27	6 (1)	46 (29)
	Anthranilate phosphoribosyl transferase	2.4.2.18	4	6
	Phosphoribosylanthranilate synthase	5.3.1.24	No hits	No hits
	Indol-3-glycerol phosphate synthase	4.1.1.48	4 (2)	6 (2)
	Trp synthase	4.2.1.20	8 (1)	30 (1)
	Phe ammonia-lyase	4.3.1.5	5	13
	Cinnamate 4-hydroxilase	1.14.13.11	1	3
	4-Coumarate coenzyme A ligase	6.2.1.12	10 (1)	40 (1)

**Flavonol biosynthesis**	Naringenin chalcone synthase	2.3.1.74	6	48
	Chalcone isomerase	5.5.1.6	3	17
	Flavanone 3-hydroxylase	1.14.11.9	2 (1)	12 (1)
	Flavonol 3-hydroxylase	1.14.13.21	6	7
	Flavonol synthase	No E.C.	2	19
	Flavonol 3-O-glucosyltransferase	No E.C.	No hits	No hits

**Flavonoid biosynthetic pathway: anthocyanin biosynthesis**	Dihydroflavonol 4-reductase	1.1.1.219	No hits	No hits
	Leucoanthocyanidin dioxygenase	NoE.C.	2	33
	UDP-flavonol 3-O-glucosyltransferase	2.4.1.-	No hits	No hits
	Anthocyanin 5-O-glucosyltransferase	NoE.C.	2	9
	Anthocyanin 5-aromatic acyltransferase	2.3.4.153	6	36
	Anthocyanin permease	NoE.C.	No hits	No hits

**Proanthocyanidin biosynthesis**	Anthocyanidin reductase	1.3.1.77	No hits	No hits
	Leucoanthocyanindin reductase	No E.C.	2 (1)	4 (2)

^a^Number of citrus unigenes annotated with the emzymatic activiy

^b^Total number of ESTs corresponding with the unigenes

The number between brackets indicates the number of unigenes (c) or ESTs (d) that are first reported in this work

**Table 5:** Lignin Biosynthesis Pathway

**Enzymatic Activity**	**Reaction EC**	**Gene Name**	**Citrus Unigenes**^a^	**Phyl. Anal.**^b^	**ESTs**
4-coumarate coenzyme A ligase	6.2.1.12	At1g51680	1	1	4
		At1g62940	1	1	5
		At1g65060	1 (1)	1	7
		At3g21240	1 (1)	1	9
		At4g05160	1	1	1
		At5g63380	3 (1)	3	13
		At1g20510	1	1	5
	
	**Total**	**7**		**9**	

5-hydroxy coniferaldehyde o-methyltransferase	2.1.1.68	At5g54160	5 (1)	5	41

caffeoyl-CoA O-methyltransferase	2.1.1.104	At4g34050	3	2	14

cinnamoyl-CoA reductase	1.2.1.44	At1g80820	1	1	2
		At2g23910	1	1	10
		At4g30470	1	1	7
		At5g14700	1	1	10
	
	**Total**	**4**		**4**	

cinnamyl-alcohol dehydrogenase	1.1.1.195	At1g09490	1	1	1
		At1g51410	1	1	2
		At4g27250	1	1	1
		At5g19440	2 (1)	2	1
		At3g19450	1	1	6
		At4g34230	1	1	1
	
	**Total**	**6**		**7**	

coniferyl aldehyde 5-hydroxylase	NIL	At4g36220	3 (2)	3	15

coumarate 3-hydroxylase	1.14.13.36	At2g40890	2	2	25

feruloyl coenzyme A reductase	1.2.1.44	At1g15950	2	2	32

UDP-glucose 4-epimerase	1.2.1.44	At2g02400	2	2	17
		At2g33590	1	1	13
		At5g58490	1	1	62
	
	**Total**	**3**		**4**	

^a ^Number *Citrus *unigenes annotated assigned to the enzymatic activty

^b ^Nimber of Unigenes that clustered with the Arabidpopsis proteins asociated with the enzymatic activity, in the phlyogenetic analysis.
