# Supplementary material for: Analysis of 13000 unique Citrus clusters associated with fruit quality, production and salinity tolerance
Source: BMC Genomics. 2007 Jan 25;8:31. doi: 10.1186/1471-2164-8-31 (PMC1796867; doi:10.1186/1471-2164-8-31)
Supplement: Additional file 1 — Putative gene duplications in Citrus. The table contains the A. thaliana loci that have been foun duplicated in Citrus. [file 1471-2164-8-31-S1.doc]

**Additional Table A. Putative gene duplications in *Citrus***

| ***At* protein** | ***Citrus* Unigenes** | **Gen Bank EST Accession nº** | **Simil.** | **Description** |
| --- | --- | --- | --- | --- |
| At5g08530 | Contig4789 | DY263182 DY274082 DY281359 DY290441 DY298453 DY303836 | 93% | NADH-ubiquinone oxydireductase 51 KDA subunit, mitochondrial |
|  | IC0AAA74CF12 | DY290172 | 71% |  |
| At5g08280 | Contig3812 | DY262715 DY263495 DY271618 DY274454 DY278145 DY280941 DY284404 DY286771 DY286902 DY290835 DY291285 DY291643 DY291936 DY293775 DY301358 DY303592 | 82% | Hydroxymethylbilane synthase / prophobilinogen deaminase, chloroplast |
|  | KN0AAQ6YP05 | DY303841 | 68% |  |
| At4g21110 | Contig4471 | DY295820 #N/A | 96% | G10 famiñy protein, contains PFAM profile: PF01125 |
|  | Contig6838 | DY264011 DY270166 DY284192 DY296838 DY302827 | 84% |  |
| At4g17760 | IC0AAA3AE07 | DY275702 | 88% | Expressed protein |
|  | IC0AAA60AG10 | DY284019 | 71% |  |
| At3g17670 | IC0AAA26BD02 | DY269216 | 78% | Ferredoxin-related, contains PFAM PF00515: TPR DOMAIN |
|  | IC0AAA39BB03 | DY275391 | 87% |  |
| At2g21150 | Contig4069 | DY262647 DY263949 DY276048 DY281531 DY283694 DY283897 DY289201 DY291023 DY292623 DY297250 DY298289 DY299341 DY305850 DY301319 DY301548 DY302733 | 82% | XAP5 family prortein, contains PFAM profile: PF04921 |
|  | KN0AAB2DD09 | DY258061 | 100% |  |
| At1g80940 | KN0AAI2CH07 | DY258631 | 82% | Expressed protein |
|  | KN0AAL3AH07 | DY306702 | 73% |  |
| At1g74470 | Contig2342 | DY275174 DY276358 DY279820 DY281092 DY285713 DY295602 DY297587 DY300948 | 82% | Geranylgeranyl-reductase |
|  | Contig4331 | DY264284 DY264561 DY273584 DY274068 DY275127 DY284153 DY287483 DY289159 DY290544 DY294757 DY297092 DY298868 DY260001 | 69% |  |
| At1g31410 | Contig3803 | DY272166 DY282062 DY288231 DY288937 DY290756 DY299278 DY270315 | 73% | Putrescine-binding periplasmic protein-related |
|  | Contig6419 | DY259283 DY259347 DY270493 | 77% |  |
| At1g29900 | KN0AAP4YA12 | DY261785 | 86% | Carbamoyl-phospahte synthase family protein A |
|  | Contig1135 | DY271706 DY274560 DY296659 DY303747 | 73% |  |
| At2g20190 | Contig1589 | DY274675 DY294835 | 77% | Clip-associating protein (CLASP)-related |
|  | IC0AAA33CF06 | DY273337 | 83% |  |
| At2g44970 | IC0AAA12CH04 | DY263779 | 68% | Lipase related |
|  | Contig1956 | DY277262 DY290283 | 77% |  |
| At3g06610 | IC0AAA53DF01 | DY281421 | 90% | DNA-binding enhancer protein-related |
|  | KN0AAB1DD08 | DY257852 | 87% |  |
| At3g58490 | KN0AAQ3YM14 | DY302731 | 78% | Phospatidic acid phosphatase family |
|  | KN0AAP9YF19 | DY271250 | 66% |  |
| At4g30790 | IC0AAA29BG10 | DY271466 | 78% | Expressed protein |
|  | IC0AAA21DG04 | DY267610 | 71% |  |
| At4g34260 | Contig6862 | DY273015 DY275135 DY287205 DY302990 DY304848 | 72% | Expressed protein |
|  | Contig0249 | DY263747 DY263929 | 81% |  |
| At5g60340 | KN0AAP2YH15 | DY261323 | 87% | MAOC-like dehydratase deomain-containing protein |
|  | KN0AAP1YH19 | DY261049 | 91% |  |
| At2g44970 | IC0AAA12CH04 | DY263779 | 68% | Lipase related |
|  | Contig1956 | DY277262 DY290283 | 77% |  |
| At3g06610 | IC0AAA53DF01 | DY281421 | 90% | DNA-binding enhancer protein-related |
|  | KN0AAB1DD08 | DY257852 | 87% |  |
| At3g58490 | KN0AAQ3YM14 | DY302731 | 78% | Phospatidic acid phosphatase family |
|  | KN0AAP9YF19 | DY271250 | 66% |  |
| At4g30790 | IC0AAA29BG10 | DY271466 | 78% | Expressed protein |
|  | IC0AAA21DG04 | DY267610 | 71% |  |
| At4g34260 | Contig6862 | DY273015 DY275135 DY287205 DY302990 DY304848 | 72% | Expressed protein |
|  | Contig0249 | DY263747 DY263929 | 81% |  |
| At5g60340 | KN0AAP2YH15 | DY261323 | 87% | MAOC-like dehydratase deomain-containing protein |
|  | KN0AAP1YH19 | DY261049 | 91% |  |
